# Neurotransmitter Regulatory Networks: A New Perspective on Cancer Therapy

**DOI:** 10.3390/biom15101429

**Published:** 2025-10-09

**Authors:** Xiaoyu Zhang, Jiaxin Cao, Yishu Zhang, Chuanxiong Li, Yuhong Jing

**Affiliations:** 1 Institute of Anatomy and Histology & Embryology, Neuroscience, School of Basic Medical Sciences, Lanzhou University, Lanzhou 730000, China; zhxiaoyu2024@lzu.edu.cn (X.Z.); caojx2021@lzu.edu.cn (J.C.); xianbaodandan@163.com (Y.Z.); lichuanxiong@kmmu.edu.cn (C.L.); 2 Key Laboratory of Preclinical Study for New Drugs of Gansu Province, Lanzhou University, Lanzhou 730000, China

**Keywords:** cancer neuroscience, neurotransmitters, receptor, neural signaling, targeted therapy, PI3K/AKT pathway

## Abstract

In recent years, the scientific community has increasingly delved into the study of the interaction between the nervous system and tumors, revealing that the nervous system not only regulates bodily functions under physiological conditions, but also assumes a vital part in the emergence and progression of tumors. Research has demonstrated that the extensive neural network directly regulates tumor progression and can influence tumors by modulating the tumor microenvironment and immune system. Moreover, tumors induce neural networks to provide favorable conditions for their proliferation and metastasis. In the above process, neurotransmitters play a vital role. They directly act or bind to their receptor, activating various classical signaling pathways, among which are PI3K/AKT, MEK/ERK, and WNT/β-catenin, to facilitate tumor advancement. Therefore, this study systematically reviews the regulatory mechanisms of neurotransmitters and their receptors in the advancement of cancer, along with the utilization of targeted drugs. At the same time, we also analyzed that targeting specific receptor subtypes may produce more significant therapeutic effects in different types of cancer. Additionally, this research further explores the limitations of neurotransmitter-based drugs currently used in clinical cancer treatment. In summary, the field of cancer neuroscience is rapidly advancing, constantly revealing the regulatory effects of neurotransmitters on tumor progression and their specific molecular mechanisms, providing broad application prospects for future clinical therapy.

## 1. Introduction

Cancer has long been a formidable challenge in the history of medical science, and its emergence and progression are intimately associated with the dysfunction of multiple systems [1]. Cancer treatment has garnered extensive attention. Despite traditional therapeutic approaches like surgical resection, radiotherapy, chemotherapy, and interventional therapy have achieved certain results, the recurrence, metastasis, drug resistance, and tumor heterogeneity of cancer, a highly complex disease, remain key factors affecting treatment outcomes. The nervous system, being the core system exerting a dominant function within the body, assumes a significant role in regulating tumor advancement and influencing cancer metastasis. Consequently, the field of cancer neuroscience has garnered considerable attention [2]. Although it is still in its infancy as an emerging research field, it is developing rapidly.

In the nervous system, signal transmission typically relies on neurotransmitters, especially those involved in rapid and precise complex signaling. As research deepens, more evidence suggests that neurotransmitters regulate cancer progression through multiple mechanisms [3,4,5]. Therefore, exploring the impact of neurotransmitters on cancer progression is beneficial for us to understand the nature of cancer from a new perspective and may bring entirely new strategies for cancer treatment. In this review, we outline the molecular mechanisms as well as the clinical applications of different types of neurotransmitters in the advancement of cancer, explore the potential of neurotransmitters as biomarkers, and emphasize therapeutic strategies of targeted drugs in cancer treatment. Through this research perspective, we hope to provide innovative theoretical and practical guidance for the field of cancer therapy.

## 2. The Biological Basis of Neurotransmitters

Neurotransmitters are a type of chemical substance responsible for signal transmission in the nervous system. As a chemical messenger, they rapidly transmit signals between neurons and between neurons and effector cells [6,7]. The classic pathway for neurotransmitter transmission is orthodromic conduction, which means that when presynaptic neurons are excited and transmitted to the axon terminals, the presynaptic membrane depolarizes, opens voltage-gated calcium ion channels, and the calcium influx facilitates the fusion of synaptic vesicles containing neurotransmitters with the presynaptic membrane. Neurotransmitters are discharged into the synaptic cleft and attach to the corresponding receptors on the postsynaptic membrane, thereby transmitting nerve impulses to the next neuron or effector [8]. Classical neurotransmitters primarily utilize the orthodromic conduction pathways to transmit signals, including acetylcholine, norepinephrine, dopamine, serotonin, glutamate and gamma-aminobutyric acid (GABA). Next is the antidromic conduction [9], which refers to the process of transmission from postsynaptic neurons to presynaptic neurons. This mode of communication is crucial for regulating synaptic plasticity, signal feedback, neuroprotection and repair, as well as the correct formation of neural circuits. The molecules that use the above mode for signal transduction are called retrograde messengers. There are mainly NO, CO, and endogenous cannabinoids. In addition, there is also a special class of high molecular weight bioactive substances called neuropeptides, which are mostly composed of 5–31 amino acid residues [10]. They can act as neurotransmitters or neuroregulatory factors, participating in various functions of the nervous system. Neuropeptides are not typical neurotransmitters, but like neurotransmitters, they act on receptors after release, and then regulate the release and function of neurotransmitters. Compared with classical neurotransmitters, neuropeptides exert broader and long-term physiological regulatory effects [11]. Different neurotransmitters require specific enzymes to catalyze their synthesis process. For example, monoamine neurotransmitters such as dopamine are converted from amino acids (such as tyrosine) [12], while amino acid neurotransmitters such as GABA and glutamate are directly derived from amino acid metabolism [13]. The site of neurotransmitter synthesis is often located in the cell body or axon end of neurons, and stored in vesicles for release.

**Figure 1:**
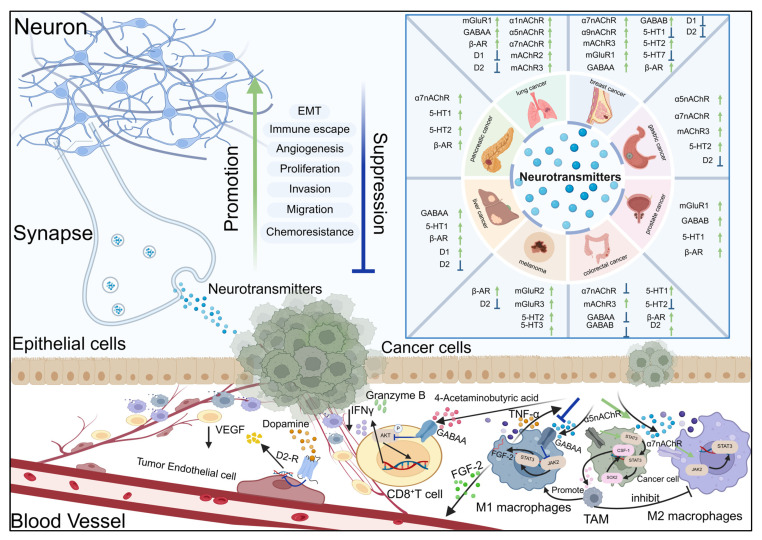
Neurotransmitter substances regulate signal transduction and the development of cancer by activating their receptors.

Recent investigations have demonstrated that neurotransmitters not only play a significant role in the nervous system, but may also be implicated in the emergence, advancement, and metastasis of cancer [14,15]. Neurotransmitters interact with tumor cells and immune cells to be involved in the modulation of cancer-related biological procedures like angiogenesis, immune escape, and cell proliferation [16]. Studies have discovered that neurotransmitters assume a dual role in cancer progression, and the promotion or inhibition of tumor progression depends on specific neurotransmitter types, receptor expression, and microenvironment factors [17]. For example, neurotransmitters such as norepinephrine accelerate angiogenesis to provide a good metastatic environment for tumor cells and promote tumor spread and metastasis [18]. However, neurotransmitters such as dopamine show inhibitory effects in some cancers, inhibiting tumor growth by slowing angiogenesis or activating the immune response [19]. Therefore, in the next section, we describe the functions and molecular mechanisms of different neurotransmitters in regulating cancer progression, as shown in Figure 1. We systematically discuss their potential as therapeutic agents. This provides more ideas for the clinic to improve the survival and prognosis of patients.

The signal interaction between neurons and epithelial cells, vascular endothelial cells, and immune cells. The figure lists the expression and activation status of key neurotransmitter receptors (including nAChR subtypes α1, α5, α7, α9, mAChR2/3, GABAA, GABAB, D1 and D2 dopamine receptors, β - AR, 5-HT1/2/3/7, mGluR1/2/3) in tumor tissues. These biological functions include epithelial–mesenchymal transition (EMT), angiogenesis, immune escape, proliferation, invasion, migration, chemoresistance, and synaptic plasticity. The green arrow (↑) indicates that receptor activation promotes biological function. The blue arrow (↓) indicates the inhibition of biological function after receptor activation. After neurons release neurotransmitters and act on tumor cells, they affect different cell types (neurons, epithelial cells, endothelial cells, immune cells, and tumor cells) through ligand receptor interactions, forming a microenvironmental signaling network that jointly regulates tumor progression and nervous system function.

## 3. The Role of Major Neurotransmitters in Cancer

### 3.1. Classic Neurotransmitters

#### 3.1.1. Acetylcholine

**Figure 2:**
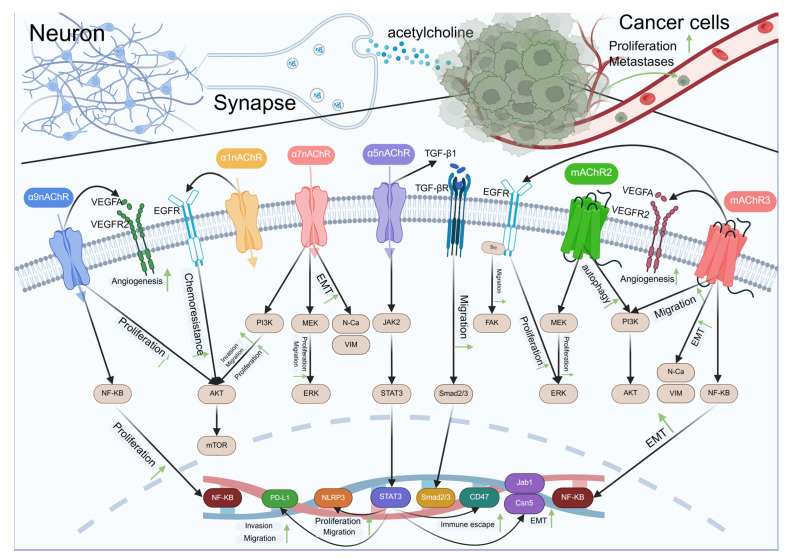
Acetylcholine-type neurotransmitters regulate cancer development via receptors.

Acetylcholine (Ach) is an ester formed by choline and acetic acid, containing quaternary ammonium ions and showing strong alkalinity. It is the chemical transmitter of cholinergic nerves and is mainly synthesized in nerve endings [20]. The synthetic pathway is that acetyl coenzyme A transfers the acetyl group to choline under the catalysis of choline acetyltransferase (ChAT). The synthesized Ach regulates various physiological processes by activating the corresponding receptors, and finally is inactivated through the hydrolysis, diffusion, and reuptake pathways of cholinesterase. ACh receptors are divided into two categories, including nicotinic acetylcholine receptors (nAChRs) and muscarinic acetylcholine receptors (mAChRs) [21]. nAChRs are a ligand-gated ion channel receptor constituted by diverse α and β subunits, including α1-10, β1-4, and α4β2, α3β4, and α6β2β3. These diverse subunit combinations result in multiple nAChRs subtypes [22]. This receptor holds a significant position in neural signal transmission, cognitive function, and the regulation of the autonomic nervous system [23]. nAChRs are not only widely present in the nervous system, but also expressed in certain non-neural tissues (like immune cells and epithelial cells). mAChRs are a G protein-coupled receptor, currently divided into five pharmacological subtypes [24], all of which signal through G protein mediation. However, the M1, M3, and M5 subtypes trigger the activation of phospholipase C (PLC) on the plasma membrane through Gq/11 proteins, hydrolyzing 4,5-bisphosphatidylinositol (PIP2) into inositol trisphosphate (IP3) and diacylglycerol (DG), stimulating the release of Ca^2+^ from the endoplasmic reticulum and increasing intracellular Ca^2+^ concentration and protein kinase C (PKC) activation. Thus, it participates in mediating cell signal transduction. M2 and M4 inhibit adenylyl cyclase (AC) through Gi protein, thereby reducing the generation of cyclic adenosine monophosphate (cAMP), and then regulating cell activities and affecting physiological functions [25]. M1 receptors are predominantly located in the cerebral cortex, hippocampus, striatum, as well as the thalamus, and are closely related to cognitive function, learning, and memory; M2 receptors are mainly distributed in the heart and are capable of regulating heart rate, and they are also distributed in nerves and smooth muscles; M3 receptors are widely distributed in exocrine glands and intestinal smooth muscles, responsible for gland secretion and smooth muscle contraction; M4 receptors are distributed in many brain regions, but its role in the striatum is particularly prominent, and it negatively feedback regulates the release of dopamine; M5 receptors are less distributed, mainly located in the ventral tegmental area and substantia nigra of the midbrain, and exert a function in modulating dopamine neuron activity as well as vascular dilation [26]. With the in-depth discussion of cancer neuroscience, studies have revealed that acetylcholine is intimately associated with the progression of various cancers. It regulates tumor progression by activating different receptor subtypes, as shown in Figure 2.

Schematic representation of the crosstalk between neurons and cancer cells mediated by acetylcholine and various signaling pathways. Neurons release acetylcholine that interacts with α7nAChR, α3nAChR, and α5nAChR receptors on the synaptic membrane. These interactions trigger downstream signaling pathways involving VEGFR2, EGFR, TGF-βR, and mAChR2/3, leading to cellular responses such as angiogenesis, chemoresistance, proliferation, and migration. In cancer cells, acetylcholine binding to mAChR3 also influences proliferation and metastasis through PI3K/AKT and NF-κB pathways. The diagram illustrates the complex interplay between neuronal signaling and cancer progression, highlighting potential therapeutic targets for cancer treatment.

Based on the latest report of the World Health Organization International Agency for Research on Cancer, lung cancer holds the position as the malignancy with the greatest mortality rate globally, featuring a mortality rate of 18.7%, and smoking constitutes a significant risk factor for lung cancer [27]. After nicotine enters the human blood circulation through the lungs, it can directly pass through the blood–brain barrier and exerting an effect on nAChRs [28]. Studies have found that nicotine is capable of upregulating HIF-1α to enhance the Warburg effect of lung cancer cells via nAChRs receptors [29]. In non-small cell lung cancer, the combination of ACh and α7nAchR activates the MEK/ERK signal and promotes the EMT development of lung cancer cells [30]. Many studies have additionally discovered that the activation of nAChRs significantly promotes the proliferation, invasion, migration, angiogenesis, chemoresistance, and immune escape of various cancer cells [31], as shown in Table 1.

This indicates that research on α5 and α7nAChR (especially lung cancer) has more opportunities to serve as a novel target for cancer therapy. In gastric cancer, the combination of Ach and mAChRM3 can activate the overexpression of nerve growth factor (NGF) in the gastric epithelium, and NGF targets the TrkA receptor to increase synaptic growth, regulate mucosal innervation, and promote tumorigenesis [56]. For patients suffering from non-small cell lung cancer, despite the fact that EGFR–tyrosine kinase inhibitors (EGFR-TKI) can be employed for treatment, the emergence of drug resistance significantly influences survival and prognosis. Researchers found that Ach mediates the development of chemoresistance by inducing the activation of the WNT signaling pathway, and blocking the Ach/mAchRM3 signal significantly reduces tumor recurrence [57]. In addition, mAChRs, including mAchRM3, also promote the proliferation, invasion, and migration of other tumors, as shown in Table 2.

Not all mAChRs can cooperate with tumor progression. Studies have found that in colon cancer, the elevated expression level of mAChRM1 is capable of suppressing the development of colon cancer; however, the precise mechanism remains unknown. Augmenting the expression of mAChRM1 might be capable of suppressing the tumor-promoting effect of other mAChRs subtypes, which may be an effective target for the therapy of colon cancer. Clinical investigations have found that the expression of mAChRM3 is extremely low in well-differentiated tumors and normal tissues, but as the degree of differentiation decreases, the expression level of mAChRM3 also continues to increase, indicating that mAChRM3 is closely related to the malignancy of the tumor [75]. This also suggests that compared with mAChRM3, mAChRM1 might exert a more crucial function in the initial phase of gastric cancer. Consequently, investigations on mAChRM1 could be more beneficial for the therapy of patients suffering from early gastric cancer.

#### 3.1.2. Glutamate

Glutamate is an excitatory neurotransmitter that can be formed by adding amino groups to α-ketoglutarate under the action of transaminases, or by deamination of glutamine by glutaminase [76,77]. Upon depolarization of nerve terminals, vesicular glutamate is released into the synaptic cleft, where it binds to specific receptors to enhance neuronal excitability. Glutamate receptors are broadly classified into ionotropic (iGluRs) and metabotropic (mGluRs) types. iGluRs include AMPA, KA, and NMDA receptors, which differ in structure, function, and activation kinetics [78]. AMPA receptors are homo- or heterotetramers (GluA1–4) widely expressed in the cerebral cortex, limbic system, and thalamus. They mediate fast excitatory postsynaptic currents, support rapid synaptic transmission, and are essential for learning and memory. KA receptors are multimers composed of five subunits, including Gluk1-3 with low affinity binding sites and GluK4 and Gluk5 with high affinity binding sites. KA receptors are extensively dispersed in the central and peripheral nervous systems, featuring an activation rate between AMPA and NMDA, a faster response, and a stronger regulatory effect. KA receptors participate in certain specific synaptic activities and contribute to synaptic plasticity by modulating the release of neurotransmitters. NMDA receptors are heteromeric complexes (GluN1, GluN2, GluN3) broadly localized in the central nervous system [79]. Their activation requires both membrane depolarization (to relieve Mg^2+^ blockade) and binding of glutamate and glycine. Unlike AMPA/KA receptors, NMDA receptor opening significantly increases Ca^2+^ permeability, leading to slow, sustained excitatory postsynaptic potentials and serving as a major source of intracellular Ca^2+^ signaling. However, excessive NMDA receptor activation may cause Ca^2+^ overload, contributing to neurodegenerative pathology. In recent years, investigations have continuously found that glutamate signals are involved in the progression of diverse cancers. mGluRs belong to G protein-coupled receptors [80], and can be classified into three types of mGluRs based on their sequence similarity, agonist potency order, and intracellular signal transduction mechanism. The first group of receptors (mGlu1/5) is coupled to Gαq protein, and stimulates IP3 receptor to trigger Ca^2+^ release through a series of pathways. The second group of receptors (mGlu2/3) and the third group of receptors (mGlu4/6/7/8) are coupled to Gαi proteins, and receptor activation inhibits AC, thereby reducing intracellular cAMP levels.

**Figure 3:**
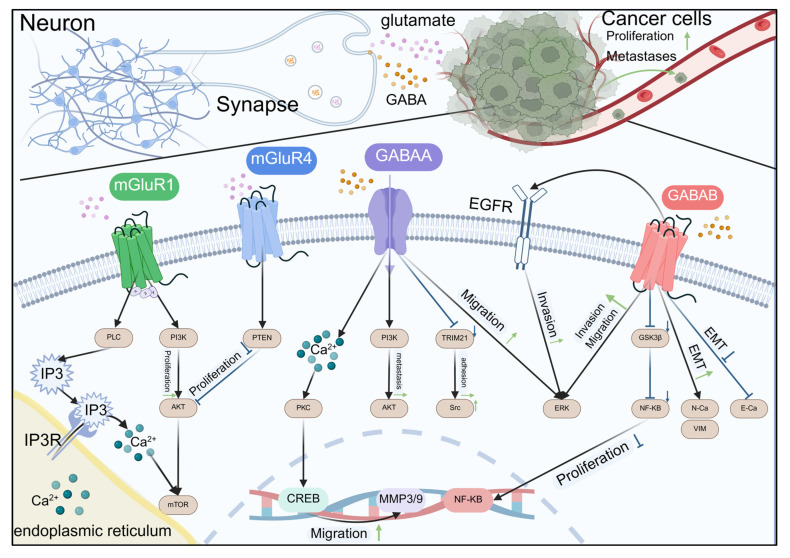
Amino acid neurotransmitters regulate cancer development through receptors.

In recent years, the research within the domain of cancer neuroscience on glutamate receptors has mainly focused on mGluRs (especially mGluR1), which have a significant role in cancer cell proliferation, invasion, migration, and angiogenesis compared to iGluRs [81,82], as shown in Figure 3 and Table 3.

Schematic representation of the intricate crosstalk between glutamate and GABA signaling in neurons and their influence on cancer cell behaviors. Neurons release glutamate and GABA that interact with mGluR1, mGluR4, GABAA, and GABAB receptors on the synaptic membrane. These interactions trigger downstream signaling pathways involving PI3K, AKT, PLC, PKC, CREB, GSK3β, NF-κB, and ERK, leading to cellular responses such as proliferation, migration, and invasion. In cancer cells, glutamate binding to mGluR1 and mGluR4 also influences proliferation and migration through PI3K/AKT pathways. GABA binding to GABAA and GABAB receptors modulates cell migration and invasion through PKC and GSK3β pathways, respectively. The diagram illustrates the complex interplay between neuronal signaling and cancer progression, highlighting potential therapeutic targets for cancer treatment.

Lung cancer is prone to brain metastasis, and the mortality rate after brain metastasis is very high, and the survival time of patients is very short, with a natural average survival time of only 1-2 months. The latest study found that lung cancer cells rely on the activation of mGluR1 signals in the brain microenvironment. This is because astrocytes secrete glutamate, which induces the mGluR1 signal in cancer cells through the WNT-5a/PRICKLE1/RE1 silent transcription factor REST axis, and then mGluR1 interacts with the epidermal growth factor receptor (EGFR) of lung cancer cells in a glutamate-dependent manner. When EGFR is stabilized by mGluR1, it enhances the migration of cancer cells, thereby accelerating the spread of cancer in brain tissue [87]. Although there are few studies on iGluRs in tumors, NMDA receptors have shown tumor-promoting effects in many cancers. Therefore, in tumor research, focusing on the regulation of NMDA receptors and mGluR1 on tumors and their mechanisms may be more helpful in developing new therapeutic strategies and biomarkers, thereby improving the efficacy of cancer diagnosis and therapy.

#### 3.1.3. Gamma-Aminobutyric Acid

Gamma-aminobutyric acid (GABA) is an inhibitory amino acid released by nerve tissue and widely present in the nervous system. GABA is generated by the decarboxylation of glutamate, stored in neurons, and released and degraded into succinic acid, which enters the tricarboxylic acid cycle, or is reuptaken by transporters to terminate the effect [88]. GABA receptors are mainly divided into two categories, GABAA and GABAB, according to their responsiveness to specific agonists and antagonists. GABAA receptors are ligand-gated ion channels that cause postsynaptic inhibition through the Cl^−^ channel, hyperpolarizing the membrane potential of neurons and reducing the excitability of neurons [89]. GABAA receptors are predominantly situated on the postsynaptic membrane, where they mediate fast inhibitory synaptic signaling within the central nervous system. GABAB receptors are a G protein-coupled receptor that inhibits AC by activating Gi/o-type G proteins and reduces intracellular cAMP levels [90]. In addition, GABAB receptors inhibit neurotransmission by regulating K^+^ and Ca^2+^ channels. In the presynaptic membrane, GABAB receptors reduce Ca^2+^ influx and regulate the release of neurotransmitters and neuropeptides; in the postsynaptic membrane, it is mainly coupled with inward rectifying K^+^ channels to mediate chronic inhibitory postsynaptic potentials. GABA is mainly distributed in the mammalian brain and serves as the primary inhibitory neurotransmitter in the brain, affecting about half of the central neurons. It is also distributed in trace amounts in other organs such as the liver, kidneys, and blood vessels.

High-grade gliomas and neural networks engage in bidirectional interactions, mutually influencing each other. Neuronal activity can increase the growth of gliomas, while gliomas also cause an increase in neuronal excitability. Therefore, the abundant GABA in the brain holds great significance for glioma research. In glioma cells, GABA is metabolized to gamma-hydroxybutyric acid (GHB), which enhances tumor cells’ energy metabolism and maintains glioma stem cell characteristics, thereby activating specific signaling pathways to promote tumor stem cell proliferation and survival [91]. Research has indicated that GABA is involved in multiple cancers, influencing cancer cell proliferation, invasion, and migration, as shown in Figure 3 and Table 4.

Various types of GABA receptors exert distinct functions in diverse cancers. In pediatric medulloblastoma (SHH-MB), after the expression of GABAA receptors is induced by agonists, it can inhibit the PKA-cAMP response element-binding protein (CREB)-Gli1 signaling pathway mediated by cAMP in SHH-MB, thereby suppressing the proliferation of tumor cells. In vivo experiments also discovered that oral administration of the GABAA receptor agonist moxidectin can notably restrain the growth of SHH-MB tumors [106], so the GABAA receptor could potentially serve as an efficacious therapeutic target. In non-small cell lung cancer, an elevated level of GABAB receptors is correlated with an unfavorable prognosis for patients. GABA inhibits GSK-3β activity by activating GABAB receptors, thereby enhancing β-catenin signaling, promoting tumor cell proliferation, and reducing CD8+ T cell infiltration. These findings imply that GABAA and GABAB receptors, as part of the GABAergic system, may act antagonistically in various cancers, just as mAchRM1 and mAchRM3 in the cholinergic system. This also reflects the high complexity between the nerve and the tumor, so it is still a major challenge in future research to clarify the specific functions and mechanisms of different receptors in different cancers.

#### 3.1.4. Norepinephrine and Epinephrine

**Figure 4:**
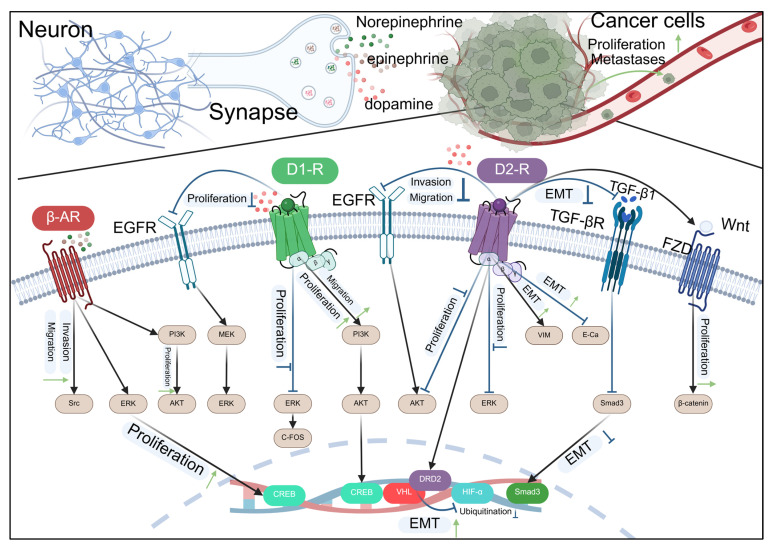
Catecholamine-type neurotransmitters regulate the development of cancer through receptors.

Norepinephrine (NE) and Epinephrine (E) are catecholamine hormones, which are synthesized from tyrosine through a series of enzymatic reactions. NE is mainly secreted in sympathetic nerve endings and the central nervous system; it functions as a neurotransmitter to convey signals across nerve synapses. Additionally, a minor quantity is released by the adrenal medulla; E is predominantly secreted by the adrenal medulla, and a small amount exists in sympathetic nerve endings. It is primarily secreted as a hormone into the bloodstream, exerting a broad spectrum of effects. Both act on adrenergic α and β receptors, but have different affinities for different receptors [107]. NE mainly acts on α1 and α2 receptors, and also has a strong affinity for β1 receptors, However, its impact on β2 receptors is weak. Consequently, it mainly affects vasoconstriction and increases blood pressure, while having a weak effect on the bronchus and metabolism. E binds strongly to α1, α2, β1, and β2 receptors, particularly β2 receptors, leading to significant dilation of the heart and bronchus [108,109]. Studies have found that NE and E, as catecholamine stress hormones, not only participate in the regulation of the cardiovascular system, but also exert a crucial function in the cancer progression, as shown in Figure 4.

This diagram illustrates the complex interactions between neurotransmitters released by neurons and signaling pathways in cancer cells. Norepinephrine, epinephrine, and dopamine, released at the synapse, can influence cancer cell behavior by interacting with various receptors. The β-adrenergic receptor (β-AR) and epidermal growth factor receptor (EGFR) pathways are activated, leading to downstream signaling through PI3K, MEK, and ERK, which promote cell proliferation and invasion. The dopamine D1 and D2 receptors also modulate cell proliferation and migration. The D2 receptor pathway involves the activation of ERK and AKT, which can lead to the inhibition of epithelial–mesenchymal transition (EMT) through the downregulation of DRD2, VHL, and HIF-α, and the upregulation of Smad3. The TGF-β receptor (TGF-βR) pathway, when activated, can induce EMT, promoting cell migration and invasion. The Wnt/FZD pathway also contributes to EMT and cell proliferation. The diagram highlights the potential therapeutic targets for inhibiting cancer cell proliferation and metastasis by modulating these signaling pathways.

It can be known from epidemiological investigations that chronic disease invasion [110,111,112,113] and long-term stress [114,115] are risk factors for cancer. Long-term stress induces hypothalamic–pituitary–adrenal axis activation, increasing cortisol levels, which indirectly promotes the release of catecholamines, forming a stress cascade effect [116]. Under chronic stress, the sympathetic nerve is constantly stimulated, and NE and E remain at high levels for a long time, driving cancer occurrence and development. The pancreas is richly innervated by sympathetic nerve fibres, and stress appears to be particularly associated with the advancement of pancreatic cancer. Studies have found that anti-stress drug treatment can decelerate the advancement of pancreatic cancer. The β-adrenergic receptor agonist isoproterenol is capable of directly facilitating the development of pancreatic tumors, whereas the non-selective β-blocker propranolol inhibits tumor growth [117]. In primary liver cancer, chronic stress promotes increased NE release and sustained activation of β-adrenergic receptors. Studies indicate that this signaling enhances HepG2 liver cancer cell proliferation via the ERK1/2/CREB axis [118] and confers survival advantages under anchorage-independent conditions—a key mechanism in metastasis [119]. E primarily signals through the β2-adrenergic receptor (β2-AR), coordinating tumor-promoting processes via downstream effectors including cAMP, PI3K/AKT, and MAPK/ERK pathways, which collectively regulate cancer cell metabolism, proliferation, and survival [120,121,122]. However, under acute stress, β2-adrenergic receptor (β2-AR) activation is transient due to rapid receptor desensitization and internalization, limiting sustained downstream signaling. Among adrenergic receptors, β2-AR plays a particularly critical role in cancer progression, largely due to its frequent overexpression in tumors and ability to coordinate multiple pro-tumorigenic processes. Upon activation, it drives key oncogenic pathways supporting proliferation, survival, and angiogenesis. It also facilitates immune evasion by suppressing the activity of T and NK cells [123,124,125] and is implicated in inflammation and angiogenesis [126]. Therefore, thoroughly exploring the mechanisms by which the β2 receptor influences cancer progression is crucial for developing targeted therapies against the β2 receptor and improving clinical management of patient.

#### 3.1.5. Dopamine

Dopamine (DA) is a catecholamine neurotransmitter, an intermediate product produced by tyrosine in the metabolic process through dihydroxyphenylalanine, and it is the precursor of NE and E synthesis. The DA system, being a crucial reward system within the human body, exerts a significant regulatory function in behaviors like emotion, learning, cognition, reward, and social interaction [127,128,129]. Dopamine receptors are categorized into two groups based on their G protein coupling and signaling properties: D1-like receptors (D1 and D5) and D2-like receptors (D2, D3, and D4) [130]. D1-like receptors are excitatory DA receptors that activate AC through the αs subunit of G proteins and enhance the excitability of downstream neurons. D2-like receptors are inhibitory DA receptors, mainly coupled with inhibitory G protein Gi/o, and inhibit AC activity and cAMP production after activating Gi/o protein, reducing neuronal excitability. It has been demonstrated by studies that the activation of dopamine receptors exerts an intricate influence on tumor proliferation, apoptosis, angiogenesis, and immune regulation, as shown in Figure 4 and Table 5.

This may be related to dopamine concentration [152,153], receptor subtype [154,155], tumor microenvironment [156,157,158], tumor type, and activated signaling pathways [157].

Generally, D2-like receptors, particularly the D2 receptor, function as tumor suppressors in many cancers by inhibiting cAMP and cyclin expression. The D2 receptor can suppress tumor growth by inducing apoptosis via p53 and caspases [159], inhibiting VEGF-mediated angiogenesis [9], and promoting anti-tumor M1 macrophage polarization [132]. However, its role is context-dependent, as it can also promote glioblastoma progression via the ERK/GSK3β/β-catenin pathway [148]. Therefore, when studying the regulation of tumor progression by dopamine receptors, the D2 receptor and different types of downstream signaling pathways may be prospective targets for the therapy of cancer. In addition, there has been an increasing amount of research on D1 receptors, and it plays different functions in different cancers. In cholangiocarcinoma, the D1 receptor can inhibit tumor growth. Inhibition of the D1 receptor increases the expression of intracellular endogenous WNT7B, thereby driving the expansion of stem cell-like populations and contributing to cholangiocarcinoma initiation and progression. Targeting the D1 receptor feedback signal could serve as a promising treatment approach for cholangiocarcinoma [145]. In contrast, in hepatocellular carcinoma, elevated DA and D1 receptor expression drives tumor proliferation and metastasis through the cAMP/PI3K/AKT/CREB pathway [139]. Therefore, in the anti-tumor treatment strategy targeting dopamine receptors, the precise selection of specific dopamine receptor subtypes and their corresponding regulators has important research and application value for the efficacy and safety of future cancer treatment.

#### 3.1.6. 5-Hydroxytryptamine

**Figure 5:**
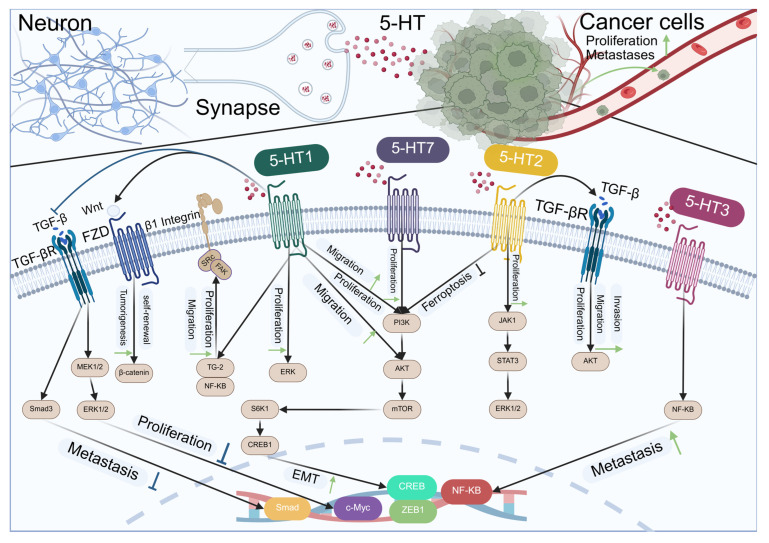
Indoleamine neurotransmitters regulate the development of cancer through receptors.

5-hydroxytryptamine (5-HT), or serotonin, is an indole ethylamine compound that cannot cross the blood–brain barrier or enter cells directly. Its synthesis is restricted to neurons. 5-HT receptors can be categorized into two principal groups, namely the ligand-gated ion channel receptor family and the G protein-coupled receptor family [160,161]. The ligand-gated ion channel receptor family includes 5-HT3A, 5-HT3B, and 5-HT3C. The G protein-coupled receptor family is categorized into three groups: receptors that couple with Gi/o proteins to inhibit AC (for instance: 5-HA1A, 5-HT1B, 5-HT1C, 5-HT1D, 5-HT1E, 5-HT1F, and 5-HT5A); receptors that couple with Gq proteins to activate PLC (such as: 5-HT2A, 5-HT2B, 5-HT2C); and receptors that couple with Gs proteins to activate AC (such as: 5-HT4, 5-HT6, and 5-HT7). 5-HT has a low content in the brain, yet it is extensively implicated in numerous physiological and pathological regulatory procedures [162]. Including food intake, body temperature regulation, sleep and wakefulness, learning and memory, drug addiction, pain, gastrointestinal diseases, and mental illness [163]. With the continuous exploration of the field of tumor neuroscience, there is evidence that 5-HT can directly promote cancer progression, or affect tumor cells proliferation, angiogenesis, invasion, and migration through various 5-HT receptors [164,165], as shown in Figure 5.

Schematic representation of the interactions between serotonin receptors (5-HT1 to 5-HT7) and their downstream signaling pathways in neurons and cancer cells. In neurons (left side), the activation of 5-HT1 receptors leads to the inhibition of proliferation and metastasis through the Wnt/β-catenin pathway, while 5-HT2 receptors promote proliferation via the ERK pathway. In cancer cells (right side), 5-HT2 and 5-HT3 receptors enhance proliferation and metastasis through the PI3K/AKT/mTOR pathway. Additionally, 5-HT7 receptors promote proliferation and migration in cancer cells via the JAK1/STAT3 pathway. The diagram also illustrates the role of 5-HT receptors in ferroptosis inhibition and the epithelial–mesenchymal transition (EMT) process, which is associated with metastasis. Key signaling molecules and pathways are indicated, including TGF-β, FZD, β1 Integrin, MEK1/2, ERK1/2, PI3K, AKT, mTOR, JAK1, STAT3, NF-κB, and the transcription factors CREB, CREB1, c-Myc, and ZEB1.

About 90% of 5-HT in peripheral tissues is synthesized in the intestine, mainly produced by enterochromaffin cells and intestinal cells in the intestine, which convert tryptophan into 5-HT and secrete it into the intestinal lumen and blood [166]. 5-HT levels within the gut lumen significantly influence the development of gastrointestinal malignancies. Research indicates that 5-HT modulates both immune functions and microbiota populations by regulating the intestinal microenvironment under physiological conditions, thereby regulating cancer progression [167]; 5-HT has been shown to exacerbate intestinal inflammation, which is a significant contributor to the occurrence of colon cancer. In contrast to normal colorectal epithelial cells, the levels of 5-HT and tryptophan hydroxylase 1 in cancer cells, animal models, and colon cancer patients are significantly up-regulated, and high levels of 5-HT enhance the stimulation of the NLRP3 inflammasome through the 5-HT3A receptor, promoting the progression of colitis-related colorectal cancer [168]. Facilitated by the serotonin transporter, 5-HT is internalized into cells, triggering the RHOA/ROCK/YAP signaling pathway, which drives the progression of colon cancer [169]. In addition, 5-HT contributes to gastric cancer occurrence by up-regulating the expression of the stemness marker LGR5 [170]. Additionally, it influences tumor progression through immune modulation, such as accelerating pancreatic cancer growth by enhancing PD-L1 levels and inhibiting CD8+ T cells infiltration [171]. 5-HT plays a significant role not only in gastrointestinal cancers but also influences other cancer types. For example, in non-small cell lung cancer (NSCLC), 5-HT triggers the c-Myc/SLC6A4 signaling pathway, enhances 5-HT reuptake in A549 lung cancer cells and establishes a positive feedback mechanism that promotes tumor metastasis [172]. Studies have shown that 5-HT not only directly acts on the development process of a variety of tumors, but also regulates tumor progression by binding to its receptors and activating downstream signals [173,174], as shown in Table 6.

Therefore, when discussing the role of 5-HT in tumor cells and its regulatory role in cancer progression, it is essential to consider its multifaceted effects. These include direct effects on tumor metabolism, heterogeneity, and the immune microenvironment, as well as the complex regulatory mechanisms that arise from its receptor binding and subsequent influence on tumor behavior. Due to the elevated level of 5-HT in the digestive system activates a variety of signaling pathways, digestive system tumors should also be the focus of attention. These findings reveal that different signaling pathways activated by 5-HT may lead to different outcomes of tumor progression, indicating the multifaceted nature of 5-HT in tumor biology and providing a new perspective for cancer treatment strategies targeting different targets.

#### 3.1.7. Retrograde Messenger

Retrograde messengers are crucial in the nervous system for enabling signal transmission from postsynaptic to presynaptic neurons, opposing the role of conventional neurotransmitters. These atypical neurotransmitters can feedback and regulate the activity of presynaptic neurons, and have important effects on synaptic plasticity, learning and memory, and the coordination and network balance between neurons [192]. Based on their functional mechanisms, retrograde messengers can be categorized into three types: the first is the transmembrane diffusible retrograde messengers, such as gaseous retrograde messengers NO, CO [193] and lipid molecule endogenous cannabinoids [194], which are the focus of current research; the second is the retrograde messengers that are released by exocytosis and act on presynaptic receptors, such as nerve growth factor, brain-derived neurotrophic factor [195], etc.; the third is those molecules that realize signal transmission through the direct interaction of postsynaptic molecules with presynaptic molecules.

#### 3.1.8. The Dual Role of NO

NO, a gaseous molecule with free radical characteristics, serves critical physiological functions across various systems, including the central nervous system. It is primarily produced from L-arginine through the catalysis of nitric oxide synthase (NOS) and interacts with soluble guanylyl cyclase (sGC). Under the activation of NO, sGC facilitates the transformation of guanosine triphosphate (GTP) into cyclic guanosine monophosphate (cGMP), initiating downstream signaling cascades involving protein kinase G (PKG) and phosphodiesterase (PDE) [196]. The functions of NO in the body are diverse and dynamic. It can interact with superoxide to form peroxynitrite, a potent oxidant, which induces cellular DNA damage. It can also directly react with DNA, causing DNA strand breaks, base mismatches, and cross-links. The accumulation of these DNA damages is one of the key factors for cell carcinogenesis [197]. Research indicates that NO significantly influences tumor progression, metastasis, angiogenesis, and chemoresistance [198,199], and shows dual roles of promoting and suppressing tumors in most cancers [200]. This dual role may stem from its concentration-dependent influence on cellular signaling pathways. In a variety of cancers, high concentrations of NO can induce apoptosis of cancer cells, while low concentrations of NO may promote tumor growth [201]. This dual role of NO suggests that we need to consider individual biological characteristics comprehensively when treating cancer to develop individualized treatment plans. In cancer treatment, precise control of the concentration and release rate of NO may exert better anti-tumor effects. In addition, post-surgical NO level monitoring can serve as a diagnostic and prognostic tool for cancer patients.

#### 3.1.9. Therapeutic Potential and Challenges of CO

CO, as an endogenous signaling molecule, also plays a vital role in biological organisms. Like NO, its primary target is sGC, which it activates to drive the conversion of GTP to cGMP. This process regulates the PKG pathway, influencing vascular dilation, stimulating phosphoprotein phosphorylation, enhancing presynaptic terminal aggregation, and ultimately modulating neurotransmitter release. Although the high affinity of high concentrations of CO with hemoglobin may cause serious health problems, at a certain concentration, carbon monoxide-releasing molecules (CORMs) have shown significant therapeutic potential in a variety of cancer models [202]. CO influences tumor behavior by suppressing cancer cell growth, inducing apoptosis, inhibiting angiogenesis, reducing drug resistance, and limiting invasion and metastasis [203,204,205,206,207]. For example, in melanoma, CO induces immunometabolic reprogramming by activating PERK-regulated protective autophagy, enhancing anti-tumor T cells activity [208]. Within the domain of breast cancer therapeutics, the ROS-activated CO prodrug can release CO at a controllable release rate, significantly inhibiting the growth of breast tumors. This research initially demonstrated both in laboratory and living systems that the speed of CO liberation significantly influences its ability to inhibit cell growth, establishing a connection between the liberation rate and growth suppression effectiveness [209]. Therapeutic intervention for pulmonary malignancies through transformation of transient reactive oxygen species (ROS) into sustained carbon monoxide delivery demonstrated significant efficacy in controlling tumor development, preventing disease relapse, and suppressing metastatic dissemination, ultimately improving patient outcomes. This transformation approach additionally improves treatment efficacy by overcoming neoplastic resistance mechanisms against reactive oxygen species-based interventions. As a gaseous signaling molecule, recent studies have found that it shows great potential in cancer treatment. The clinical application of CO mainly focuses on its combination with other treatment methods. The combination not only enhances the therapeutic effect but also reduces the dose of single treatment to reduce the toxic side effects of CO. Advancing understanding of carbon monoxide’s pharmacological mechanisms has facilitated significant progress in prodrug development and controlled-release technologies, resulting in more cost-effective cancer therapeutic approaches that offer enhanced clinical and economic benefits. However, despite the progress in laboratory research, CO still seriously affects the human blood system and nervous system, and the clinical application of CO still requires more preclinical studies and clinical trials to ensure its effectiveness and safety in clinical applications. In conclusion, although CO has great potential in treating cancer, its side effects on the body cannot be ignored. The development of tumor-selective carbon monoxide delivery systems with minimized systemic toxicity and optimized therapeutic efficacy represents a crucial focus for advancing CO-based anticancer strategies.

#### 3.1.10. Complex Regulation by Endocannabinoids

Endocannabinoids are important diffusible retrograde signaling molecules in the body, mainly including N-arachidonoylethanolamine (AEA) and 2-arachidonoylglycerol (2-AG). Cerebral endocannabinoid signaling is initiated through type I metabotropic glutamate receptor stimulation, triggering elevated intracellular calcium levels in postsynaptic neuronal compartments. PLC is activated, which in turn generates DAG, and finally 2-AG is generated. At the same time, Ca^2+^ acts on phospholipids, activates acyl transferase, cleaves to generate N-arachidonoyl phosphatidylethanolamine, and is finally cleaved by phosphodiesterase to generate AEA. Following their synaptic release, these endogenous cannabinoids exert their biological effects through presynaptic CB1 and CB2 receptor modulation [210]. CB1 receptors are predominantly localized in neural tissues, and after binding to endocannabinoids, they reduce the release of neurotransmitters by activating Gi/Go proteins and inhibiting the AC-cAMP-PKA pathway. In contrast, CB2 receptors have a lower homology with CB1 receptors, about 44% [211]. They are primarily expressed in peripheral tissues (e.g., spleen, immune cells, and tonsils) and regulate signal transduction through the inhibition of AC and N-type Ca^2+^ channels, similar to CB1 receptors [212]. in short, endocannabinoids regulate the release of neurotransmitters by affecting Ca^2+^ signaling and phospholipid metabolism, and affect neural signal transmission through CB1 and CB2 receptors [213]. CB1/2 receptors primarily exert anti-tumor effects during cancer progression. Their activation suppresses cancer cell proliferation, promotes apoptosis, and inhibits angiogenesis by modulating multiple signaling pathways [214]. The activation of CB1 receptors inhibits nerve growth factor (NGF)-induced proliferation of breast cancer cells in breast cancer [215]. Additionally, CB1 agonists ACPA and ACEA significantly suppress MDA-MB-231 breast cancer cells proliferation and induce apoptosis [216]. In bladder cancer, the CB2 agonist JWH0151 inhibits tumor growth by blocking the AKT signaling pathway [217]. However, CB1/2 receptors activation may also promote cancer progression through other signaling pathways. For example, 2-AG activates the Fyn/ERK/AP-1 signaling pathway in JB6 P+ tumor-sensitive epidermal cells through CB1/2 receptors, and this is intimately associated with tumor proliferation, invasion, metastasis, and chemoresistance [218]. Compared with CB1 receptors, CB2 receptors primarily affect tumor progression through immune regulation. For example, after AEA activates CB2 receptors, it promotes tumor progression by inhibiting the JAK1/STAT1/3 signal of tumor-killing T cells and suppressing anti-tumor immunity [219]. To sum up, CB1/CB2 receptors play a role in tumor development, but whether their activity promotes or inhibits tumor growth may vary depending on the environment. Given the anti-tumor effects of CB1/CB2 receptors in various cancers, targeting these receptors holds significant therapeutic potential. However, the central nervous system activity of CB1 receptors needs to be carefully considered, including its potential side effects such as drug addiction, tolerance, and metabolic disorders, and the immune regulation mediated by CB2 receptors cannot be ignored. Therefore, the combined application strategy of CB1/2 receptors may reduce the drug dose, reduce the side effects of single-target treatment, and may produce a synergistic effect and enhance the therapeutic effect.

#### 3.1.11. Neuropeptide

Neuropeptides are short-chain amino acid-based bioactive molecules involved in multiple physiological functions, including pain modulation, metabolic control, hormonal balance, and emotional management [220]. Different from classical neurotransmitters, neuropeptides are initially produced in neuronal cell bodies as large precursor proteins, which are subsequently transported to nerve endings via axonal transport and processed post-translationally to generate their active forms. These neuropeptides are stored in large dense-core vesicles (LDCVs), and release depends on calcium ions and voltage-gated calcium channels. This process typically requires intense stimulation, resulting in prolonged release, extended duration of action, and broad physiological effects [221]. Neuropeptides regulate tumor cell proliferation, invasion, angiogenesis, tumor microenvironment, and immune escape by modulating various signaling pathways, including MAPK, cAMP, and PI3K/AKT, mediating cancer progression.

#### 3.1.12. Neuropeptide Y (NPY) in Tumor Progression and Therapy

NPY is strongly linked to cancer progression, influencing tumor cell growth, invasion, migration, and angiogenesis through interactions with its receptors [222]. For instance, in breast cancer, upregulation of Y1 and Y5 receptors correlates with enhanced tumor cell proliferation and migration [223], while their antagonists demonstrate significant tumor-suppressive effects [224]. The Y5 receptor also promotes tumor angiogenesis by stimulating VEGF secretion [225]. In prostate cancer, NPY inhibits the apoptosis of tumor cells through NF-κB, mediating the chemotherapy resistance of the tumor [226]. In hepatocellular carcinoma, dipeptidyl peptidase 4-mediated cleavage of NPY amplifies Y5 receptor signaling, contributing to tumor progression [227]. The overexpression of NPY and its receptors in a variety of cancers provides a new research direction for targeted therapy. Studies have shown that DOX-P18, an NPY analog delivered via nanocarriers, achieves precise tumor targeting and shows potential in inhibiting growth and metastasis in triple-negative breast cancer (TNBC) [228]. Therefore, targeting NPY and its receptors shows significant promise for advancing cancer therapeutics, with potential applications in both research and clinical applications.

#### 3.1.13. Substance P Mediated Oncogenic Signaling

Substance P, a tachykinin family member encoded by the preprotachykinin A gene, significantly influences cancer progression by binding to its specific receptor, neurokinin-1 receptor (NK-1R) [229]. In breast cancer, substance P interacts with NK-1R, inducing apoptosis in a subset of NK-1R-high cancer cells. The released single-stranded RNA (ssRNA) subsequently activates adjacent tumor TLR7, facilitating metastasis [230]. Substance P also enhances cancer cell invasion and migration through the activation of NF-κB, MAPK, and other signaling pathways [231]. Therefore, substance P regulates multiple signaling pathways related to cell survival, drug efflux, tumor microenvironment, and oxidative stress by activating the receptor NK-1, mediating resistance demonstrated by cancer cells against chemotherapeutic agents [232,233].

Emerging evidence indicates that, alongside NPY and substance P, additional neuropeptides—including growth hormone-releasing peptide (GHRH) [234,235], corticotropin-releasing hormone (CRH) [236,237], and calcitonin gene-related peptide (CGRP) [238,239]—play a role in tumorigenesis. These peptides influence cancer initiation and progression through interactions with their corresponding receptors. To sum up, the regulation of cancer progression by neuropeptides shows a considerable level of intricacy and diversity, which is related to its biosynthesis, receptor activation, signal transduction, and impact on the tumor microenvironment. In-depth exploration of the regulatory mechanism of neuropeptides on cancer not only enhances our understanding of tumor biology but also provides a scientific basis for creating novel therapeutic approaches. Therapeutic strategies focusing on neuropeptides and their receptors may revolutionize cancer treatment, offering enhanced clinical benefits and a higher quality of life for patients.

## 4. Conclusions, Challenges, and Future Directions

During the innervation of tumors, neurotransmitters, as key signal transduction molecules, and their receptors are widely involved in cancer progression. Therefore, drugs based on neurotransmitters, receptors, or their related pathways are gradually receiving attention, and some neuromodulatory drugs have been explored or applied in cancer treatment. Research has identified several nAChR blockers, including α-bungarotoxin, α-cyclophosphamide, and the glycoprotein of the rabies virus, that effectively suppress nicotine-driven growth, enhance cell death, and reduce the movement of A549 lung cancer cells. These findings offer a novel approach to lung cancer therapy. In addition, mAChRs antagonists, such as Darifenacin and Atropine, also exhibit broad-spectrum anti-tumor activity across multiple cancer types. Darifenacin, a selective mAChRs antagonist, is clinically used to treat overactive bladder, whereas Atropine, a non-selective M receptor inhibitor, is widely employed to alleviate smooth muscle spasms, reduce glandular secretion, and manage bradyarrhythmias. Research indicates that both agents suppress tumor cell proliferation, invasion, and migration by targeting multiple mAChR subtypes. Propranolol, a non-selective β-adrenergic receptor antagonist targeting both β1 and β2 receptors, is widely utilized in adjuvant cancer therapy due to its ability to suppress angiogenesis, tumor cell proliferation, and invasion across various cancer types. Additionally, selective β1 blockers like metoprolol and atenolol, as well as non-selective agents such as carvedilol, have demonstrated potential in inhibiting tumor growth in lung and breast cancers. Among DA receptors, the D2 receptor has always been the focus of attention of researchers, and it plays different regulatory roles in the progression of different cancers. Two D2 receptor agonists, bromocriptine and cabergoline, suppress tumor progression through hormonal modulation and inhibition of angiogenesis associated with cancer development. Chlorpromazine, haloperidol, and sulpiride, as D2 receptor antagonists, also show anti-cancer potential by blocking the D2 receptor. In the study of 5-HT receptors, the study of 5-HT2A receptors is expected to be applied to the clinical treatment of cancer earlier. For example, the drugs clozapine and ritanserin, which are used in the clinical treatment of schizophrenia, can reduce the release of VEGF, promote the polarization of tumor-associated macrophages from the M2 to the M1 phenotype within the tumor microenvironment, thereby enhancing the anti-cancer immune response by antagonizing 5-HT2A receptors. In cancer treatment, 5-HT3 receptor antagonists such as ondansetron and granisetron are primarily used to manage chemotherapy-induced nausea and vomiting. By reducing overstimulation of the gastrointestinal tract and the brainstem vomiting center, these drugs significantly enhance patients’ quality of life, although they do not directly target cancer. High concentrations of NO and CO, while toxic to the central nervous and respiratory systems, demonstrate considerable promise for applications in cancer therapy. These gas prodrugs are precisely delivered to the tumor site through oral administration or nanoparticle technology to release appropriate concentrations of NO and CO, thereby inducing tumor cell cycle arrest, promoting apoptosis, and enhancing the sensitivity of tumor cells to radiotherapy and chemotherapy, significantly improving the therapeutic effect of tumors. Therefore, precise targeting of different neurotransmitters, receptors, and their signaling pathways provides a new perspective for cancer treatment.

Although neurotransmitters show great potential in cancer treatment, translating their research results into clinical applications still faces major challenges. First, the broad presence of neurotransmitters and their receptors throughout the body means that targeting them with agonists or antagonists could impact normal tissues, causing serious side effects. For example, β-blockers act on the entire sympathetic nervous system, and while acting on tumor cells, they may also affect the heart and blood pressure. Therefore, improving the targeting of drugs is an urgent problem to be solved to avoid the impact of neurotransmitter drugs on normal physiological functions. Secondly, neurotransmitters have multiple receptor subtypes, and their mechanism of action is complex and diverse, which increases the difficulty of treating specific subtypes. In addition, long-term use of neurotransmitter receptor antagonists may lead to tumor resistance, a major contributor to poor prognosis in advanced cancer patients. Therefore, how to avoid or reduce resistance is also an important challenge. While basic research shows the great potential of neurotransmitter drugs in the treatment of cancer, their efficacy remains insufficiently validated in human trials, with limited clinical evidence suggesting a prolonged timeline for clinical translation.

Recent studies have increasingly focused on the role of neurotransmitter–tumor microenvironment interactions. Neurotransmitters influence both tumor cells and vascular endothelial cells within the microenvironment, while also directly modulating immune cells to impact cancer progression. Furthermore, the interaction between the nervous system and tumors involves bidirectional communication, rather than unidirectional regulation. Although the specific molecular mechanism has not been fully elucidated, in-depth exploration of the neuro-tumor axis in future research will help us reveal the complex mutual regulatory network between the nerve and cancer, which holds great significance for the advancement of cancer treatment strategies. At the same time, in current clinical practice, neurotransmitter-targeted drugs are mainly used to relieve postoperative nausea and pain symptoms in cancer patients, or to enhance the sensitivity of patients to radiotherapy and chemotherapy, while the vast majority of drugs that directly act on tumors are still in the development and clinical trial stage. These drugs usually lack tumor specificity and may still cause serious side effects due to the non-specificity of the drug and individual differences in the human body at an effective dose. Therefore, in-depth research on neurotransmitter types and their receptor subtypes is crucial for the development of more efficient and precise neurotransmitter regulatory drugs, which are expected to directly act on tumors, thereby improving the effect of cancer treatment. In conclusion, the regulation of cancer progression by neurotransmitters represents a critical area of tumor neuroscience research, offering not only profound insights into tumor biology but also promising potential for clinical translation.

## Figures and Tables

**Table 1:** Regulatory mechanisms of nicotinic acetylcholine receptors in cancer progression.

Cancer	Receptor	Model Materials	Mechanism	Tumor Impact	Targeted Therapy
Lung Cancer	α1nAChR	PC9, HCC827 lung cancer cells, BALB/cAJc1-nu/nu mice.	α1nAChR increases the resistance of non-small cell lung cancer to erlotinib by activating the EGFR/AKT/ERK pathway [32]	Chemoresistance	
	α5nAChR	Samples from patients with lung adenocarcinoma; A549, H1299, NCI-H1975, PC9, H226, and HCC827 lung cancer cells; lung tumor xenograft BALB/c nude mice; chicken embryo chorioallantoic membrane (CAM) model.	α5nAChR promotes EMT by regulating Stat3-Jab1/Csn5 [33]. α5nAChR mediates the STAT3/PD-L1 signal, promoting tumor invasion and migration [34]. α5nAChR mediates the AKT pathway to down-regulate JWA expression and thereby induces lung cancer stemness and progression [35]. α5nAChR up-regulates CD47 through STAT3 to facilitate immune escape [36]. nicotine activates the α5nAChR/SOX2/CSF-1 axis to promote M2 macrophage polarization and thereby inhibits the immune response [37]. α5nAChR promotes non-small cell lung cancer migration through the TGF-β1/Smad signal [38]. low-dose nicotine activates the EGFR signal through α5nAChR, increasing the levels of mesenchymal markers such as N-Ca, Slug, and VIM, and promoting the invasion and migration of lung adenocarcinoma cells [39]. α5nAChR mediates NLRP3 expression, and nicotine activates the α5nAChR/STAT3/NLRP3 axis to promote the proliferation and migration of lung cancer [40].	Proliferation EMT Invasion Migration Stemness Immune Escape Immunosuppression	JAC4 [35]
	α7nAChR	H460, h1975, A549 lung cancer cells, Lewis mouse-derived lung cancer cells.	Qnd7, an α7nAChR antagonist, inhibits the proliferation and migration of lung cancer cells by inhibiting AKT/mTOR signaling [41]. Sinomenine inhibited α 7nachr and related signaling molecules pERK1/2 and transcription factors TTF-1 and SP-1, and decreased the proliferation and migration ability of lung cancer A549 cells [42].	Proliferation Migration	APS8-2 [43] QND7 [41] Sinomenine [42]
Gastric cancer	α5nAChR	MKN28, BGC823, MGC803, AGS gastric cancer cells; gastric cancer patient samples.	5nAChR is highly expressed in gastric cancer tissues, and activates AKT to promote cancer cell proliferation and cisplatin resistance [44].	Proliferation Chemoresistance	
	α7nAChR	BGC, SGC gastric cancer cells; Xenograft tumor female nude mice.	α7nAChR enhances the phosphorylation level of MEK/ERK and promotes the development of EMT, while the use of RL RVG can inhibit this process [45].	Invasion Migration	rL-RVG [45]
Breast cancer	α7nAChR	MCF-7, T47D MDA-MB-435, MDA-MB-231 breast cancer cells.	Nicotine acts on α 7nAChR to activate fibroblasts and promote EMT and migration ability of breast cancer cells [46].	Migration EMT	
	α9nAChR	breast cancer cell lines.	αO-conotoxin GeXIVA, a selective antagonist of α9nAChR, can induce cancer cell apoptosis, inhibit cell proliferation mediated by AkT mTOR, STAT3 and NF-κB, and inhibit tumor growth in vivo [47]. α9nAChR mediates the proliferation of breast cancer cells, and α o-conotoxin gexiva can inhibit this process [48,49]. Nicotine promotes VEGF-A, VEGFR2, and p-vegfr2 expression through α9nAChR, promoting angiogenesis, migration, and proliferation [50].	Proliferation Apoptosis Migration Angiogenesis	αO-conotoxin GeXIVA [1,2,47] αO-Conotoxin GeXIVA [48,49] α9 BsAb [50] MEC [51]
Colorectal cancer	α7nAChR	Lovo colorectal cancer cells.	α7nAChR inhibited the invasion and migration of human colon cancer cells through PI3K/AKT signaling pathway [52]. JAK2/STA3 was inhibited and the migration ability of cancer cells was weakened afterα7nAChR knockdown in tumor associated macrophages [53].	Invasion Migration	
Pancreatic cancer	α7nAChR	HPNE pancreatic epithelial cells and Capan1 pancreatic cancer cells; KrasG12D, PDX1 CRE (KC) mice.	Cigarette smoke promotes the expression of Paf1 gene through α7nAChR-ERK-FOSL1 signaling pathway, while increasing PHF5A levels. PAF1 and PHF5A interact to induce cancer cell stemness characteristics [54].	Stemness	
Cholangiocarcinoma	α7nAChR	QBC939 and RBE human cholangiocarcinoma cells; shRNAα7nAChR, BALB/c female nude mice.	Overexpression of α7nAChR can inhibit cancer cell apoptosis, accelerate the EMT process and thus promote cholangiocarcinoma progression [55].	Apoptosis Invasion Migration	

**Table 2:** The regulatory mechanisms of mAChRs in cancer progression.

Cancer	Receptor	Model Materials	Mechanism	Tumor Impact	Targeted Therapy
Lung cancer	mAChR2	A549, PC9 lung cancer cells; xenograft tumor nude mice.	mAChRM2 activation increases MAPK and AKT phosphorylation, which promotes lung cancer cell proliferation and accelerates EMT. mAChRM2 antagonist Methoctramine was able to reverse this process of EMT [58]. The mAChRM2/ERK/AKT/NF-κB axis promotes EMT in non-small cell lung cancer [59].	Proliferation EMT	Methoctramine [58]
	mAChR3	A549, H520 lung cancer cells.	The plant base Arecoline acted as an M3 agonist and activated the EGFR/Src/FAK pathway through mAChRM3, which promoted the migration of cancer cells [60]. ACh increased IL-8 levels, induced EGFR activity through M3R, and stimulated the activation of PI3K/AKT to enhance proliferation, invasion, and migration of non-small cell lung cancer cells [61].	Proliferation Invasion Migration	
Gastric cancer	mAChR3	GES-1 gastric normal epithelial cells, MKN-28, MKN-45, BGC-823, MGC-803, SGC-7901 gastric cancer cells; BALB/c nude mice.	mAChR3 knockdown arrested the G2/M phase, inhibited the formation of GC xenograft tumors, and promoted cell apoptosis. ACh activates EGFR signaling through machrm3, induces ERK1/2 and AKT phosphorylation, and promotes cell proliferation [62]. mAChR3 antagonists 4-DAMP and darifenacin could significantly inhibit tumor formation. 4-DAMP also enhanced the toxicity of 5-FU to cells and induced apoptosis related proteins such as Bax and caseinase-3 to promote gastric cancer cell apoptosis [63]. ACh promotes GC cell invasion/migration through mAChR3/AMPK/MACC1 signaling pathway, an oncogene associated with metastasis and overexpressed in gastric cancer [64].	Proliferation Apoptosis Angiogenesis Chemoresistance	4-DAMP [62] Darifenacin [62]
Breast cancer	mAChR3	MCF-7, MCF-10A human-derived breast cancer cells, LMM3 mouse-derived breast cancer cells; three-month-old female BALB/c nude mice.	Immunoglobulin (GT1N0Mx-IgG) in clinical stage I (T1N0Mx stage) breast cancer patients increases MMP9 activity and promotes tumor cell migration through mAChRM3 [65]. T1N0Mx-IgG and carbachol promote VEGF-α production and promote neovascularization through mAChR activation [66].	Angiogenesis Migration	Atropine [66,67]
Colorectal cancer	mAChRs	H508, HT29, snu-407 colorectal cancer cells.	Hijiki and sodium arsenite induced the activation of EGFR and ERK, and atropine, an inhibitor of mAChRs, partially inhibited the activation of EGFR/ERK, which in turn inhibited the proliferation of colorectal cancer cell [68]. In SNU-407 colon cancer cells, mAChRs mediated S6K1 activation through the PI3K/AKT/mTOR1 pathway, which in turn promoted tumor cell proliferation [69]. In SNU-407 colon cancer cells, mAChRs also regulate eIF4B phosphorylation through ERK1/2 and PKC signaling pathways to promote the proliferation and migration of tumor cells [70].	Proliferation Migration	Atropine [68]
Cholangiocarcinoma	mAChR3	HuCCA-1, RMCCA-1, FRH0201, RBE human-derived cholangiocarcinoma cells; tissue samples from cholangiocarcinoma patients, and normal tissue samples from gallbladder and liver transplant patients.	The expression of mAChR3 was significantly upregulated in cancer tissues. Migration of cancer cells, infiltration of peripheral nerves and EMT progression were promoted by activation of the AKT signaling pathway. The use of the mAChR agonist pilocarpine significantly enhanced this process, while the mAChR antagonist atropine effectively inhibited mAChR activation, which in turn inhibited cancer cell migration, nerve infiltration, and EMT [71]. Taurocholic acid (TLCA) induces cholangiocarcinoma cell proliferation through the mAChR and EGFR/EKR1/2 signaling pathways, and the use of atropine inhibits cholangiocarcinoma cell proliferation by inhibiting the mAChR and thereby inhibiting cholangiocarcinoma cell proliferation [72].	Migration EMT proliferation	Atropine [71,72]
Glioblastoma	mAChR2	U251 and GB7 human glioblastoma cells.	The mAChR2 agonist N8-Iper promotes the level of autophagy in glioblastoma. mAChR2-regulated autophagy in cancer cells can be achieved by inhibiting the PI3K/AKT/mTOR signaling pathway and increasing p-AMPK levels [73].	Proliferation Autophagy	
Ovarian cancer	mAChR2	iOSE-120, iOSE-398 human normal ovarian epithelial cells, TOV-21G, SKOV-3 human ovarian cancer cells.	The mAChR2 agonist APE inhibited ovarian cancer cell proliferation, promoted apoptosis, and enhanced the sensitivity of cancer cells to chemotherapeutic drugs. Experimental data showed that APE treatment significantly elevated the proportion of G2/M-phase cells and mitotic abnormalities and improved drug response [74].	Proliferation Apoptosis Chemoresistance	APE [74]

**Table 3:** Regulatory mechanisms of mGluRs in cancer development.

Cancer	Receptor	Model Materials	Mechanism	Tumor Impact	Targeted Therapy
Breast cancer	mGluR1	HDEC vascular endothelial cells, 4T1-12B murine-derived breast cancer cells, MDA-MB231 human-derived breast cancer cells, 6- to 8-week-old female BALB/c mice.	mGluR1 signaling promotes vascular endothelial cell growth, which together with VEGF activates the downstream active substance PKC, which in turn promotes tumor growth and angiogenesis [83].	Angiogenesis Proliferation	
Prostate cancer	mGluR1	LNCaP, 22Rv1 and PC3 human-derived prostate cancer cells, LL2 murine-derived lung cancer cells C57BL/6J nude mice.	rostate-specific membrane antigen (PSMA) carboxypeptidase activates mGluR1 via glutamate release and PI3K-p110β phosphorylation and further promotes tumor growth and cell survival [84].	Proliferation	
Melanoma	mGluR2/3	Primary MDSC, B16-F10 murine-derived melanoma cells, CD4+, CD8+ T cells; C57BL/6J mice.	High expression of mGluR2/3 on myeloid-derived suppressor cells MDSC attenuated the immunosuppressive activity of MDSC and inhibited the growth of B16-F10 melanoma in vivo using the mGluR2/3 antagonist LY341495 [85].	Immunosuppression Proliferation	LY341495 [85]
Bladder cancer	mGluR4	SV-HUC human ureteral epithelial cells, RT4, T24, 253J, J82, 5637 and UMUC3 human-derived bladder cancer cells; 5-week-old male thymus-free BALB/c nude mice.	Activation of mGluR4 down-regulated cAMP/PTEN/AKT signaling, which inhibited the proliferation of bladder cancer cells, decreased the Bcl-2/Bax ratio, and promoted apoptosis of cancer cells [86].	Proliferation Apoptosis	VU0155041 [86]
Lung cancer	mGluR1	WM266.4-Luc-mEGFP melanoma cells, PC9-Luc-mEGFP human lung cancer cells; 8-10-week female BALB/c nude mice.	Astrocytes activate mGluR1 signaling in cancer cells through the Wnt-5a/PRICKLE1/REST axis, which in turn promotes the glutamate-dependent interaction of mGluR1 with EGFR, enhances the migratory ability of lung cancer brain metastatic cells, and accelerates intracerebral spread [87].	Invasion Migration	LY456236 [87]

**Table 4:** Regulatory mechanisms of GABA receptors in cancer progression.

Cancer	Receptor	Model Materials	Mechanism	Tumor Impact	Targeted Therapy
Lung cancer	GABAA	LLC murine-derived Lewis lung cancer cells, L929 murine-derived fibroblasts, Raw264.7 murine-derived monocyte macrophage leukemia cell line; 6–8-week male C57BL/6J mice; Lung cancer patients undergoing radical resection; NCI-H1975 and Lewis lung cancer cells, Beas-2B normal epithelial cells; 18–24-month-old C57BL/6 mice.	GABA released by tumor cells inhibits M1-type macrophage polarization through NF-κB and STAT3 pathways and activates STAT6 pathway to promote M2-type polarization, which in turn suppresses the immune response. In addition, GABA increases FGF2 expression in macrophages and promotes tumor neovascularization. The application of GABAA receptor inhibitors significantly reduces tumor burden [92]. Isoproterenol is able to regulate the Th17/Treg balance in perioperative lung cancer patients via GABAA receptor, which in turn inhibits the invasion and migration of lung cancer cells [93]. However, isoproterenol also enhances tumor cell adhesion and extension through the GABAAR-TRIM21-Src signaling pathway, which in turn promotes lung cancer metastasis [94].	Immunosuppression Angiogenesis Invasion Migration	Picrotoxin [92] Propofol [93]
Breast cancer	GABAA	MCF-7, BT549, MDA-MB-453, MDA-MB-436 human-derived breast cancer cells, PY8119 murine-derived breast cancer cells; breast cancer patient tissue samples; (Gpt2fl/fl) C57BL/6 mice.	Glutamine pyruvate transaminase (GPT2) activates the GABAA receptor via GABA, leading to the opening of the associated Ca^2+^ channels and an increase in Ca^2+^ endocytosis. This process further activates the PKC-CREB signaling pathway, and CREB upregulates pro-metastasis-related genes such as MMP9 to accelerate metastasis [95]. GABAA receptor isoform α3 promotes the development of lung metastasis in breast cancer cells through activation of the AKT pathway [96]. Knockdown of GABAA β3 subunit resulted in downregulation of cyclinD1 expression and upregulation of p21 expression, triggering cell cycle arrest. And knockdown of GABAA β3 inhibited cancer cell proliferation and migration [97]. The GABA receptor subunit GABRP promotes the migration of basal-like breast cancer cells through phosphorylation of ERK1/2 [98].	Proliferation Invasion Migration	
	GABAB	MCF-7 human-derived breast cancer cells, 4T1 mouse-derived breast cancer cells.	Baclofen (GABAB receptor agonist) promotes breast cancer invasion, migration, and metastasis in vivo through the ERK1/2 pathway [99].	Invasion Migration	CGP55845 [99]
Colorectal cancer	GABAA	LOVO, HT29, SW1116 human colorectal cancer cells.	Propofol significantly reduced MMP2 and MMP9 levels through GABAA receptors and inhibited ERK1/2 phosphorylation thereby reducing cancer cell invasion [100].	Invasion	
	GABAB	RKO, DLD1, Lovo, HCT116, HT29, SW620 human-derived colorectal cancer cells; 5-week BALB/c nude mice; HT29, 5-FU-resistant HT29 human-derived colorectal cancer cells.	GABABR1 reduces colorectal cancer cell invasion and migration by inhibiting the EMT and Hippo/YAP1 pathways [101]. GABAB receptor activation inhibits GSK-3β activation and thus NF-κB, which inhibits colorectal cancer cell proliferation [102]. GABAB receptors suppress cancer cell metastasis and induce 5-FU-resistant cell apoptosis through inhibition of the cAMP-dependent signaling pathway and the inhibitor of apoptosis protein 2 (cIAP2) to inhibit cancer cell metastasis and induce apoptosis in 5-FU-resistant cells [103].	Invasion Migration EMT Proliferation Apoptosis Chemoresistance	Baclofen [102]
Prostate cancer	GABAB	PC-3 human-derived prostate cancer cells.	GABAB receptor selectively induces EGFR activation which in turn mediates ERK1/2 activation and further promotes prostate cancer cell invasion and migration [104].	Invasion Migration	
Liver cancer	GABAA	HEK293 human embryonic kidney cells, MHCC97L and Hep3B human-derived hepatocellular carcinoma cells, Hepa1-6 murine-derived hepatocellular carcinoma cells, 4-week-old male BALB/c nude mice, PMVK^fl/fl^ mice, Alb-Cre mice, tissue samples from patients with hepatocellular carcinoma.	In hepatocellular carcinoma, phosphomercuric acid kinase (PMVK)-mediated 4-acetylaminobutyric acid (4-AC-GABA) activates GABAA receptors on CD8+ T cells in the tumor microenvironment and further inhibits CD8+ T cell activation, intra-tumor infiltration, and anti-tumor responses by inhibitingAKT signaling [105].	Immune escape	

**Table 5:** Regulatory mechanisms of dopamine receptors in the progression of cancer.

Cancer	Receptor	Model Materials	Mechanism	Tumor Impact	Targeted Therapy
Breast cancer	D1	4T1 murine breast cancer cells, MDA-MB-231 human breast cancer cells; 5-week female BALB/c mice.	D1 receptor knockdown promotes breast cancer cells to undergo invasion and lung metastasis, and the oral compound QAP14 was able to activate D1DR to inhibit stem cell properties and EMT metastasis in breast cancer [131].	EMT Metastasis	QAP14 [131]
	D2	MDA-MB231, BT549, YCCB1, 4T1 breast cancer cells, MCF-10A, HMEC mammary epithelial cells, THP-1 cells; 6–8- week BALB/c mice, 6-week female BALB/c mice. B16-F10 mouse melanoma cells, 4T1 mouse breast cancer cells; 4–6-week female C57BL/6J mice.	D2 receptor inhibits EMT progression in breast cancer cells; promotes macrophage polarization to M1-type in the tumor microenvironment while downregulating IL-6 and IL-10; and downregulates DDX5 and eEF1A2 to inhibit the NF-κB pathway [132]. Hypermethylation of the D2 receptor promotes proliferation, migration, and tumor growth of breast cancer through the FLNA-ERK pathway [133]. The D2 receptor antagonist thioridazine hydrochloride (Thioridazine hydrochloride) inhibits the proliferation and migration of triple-negative breast cancer cells by inhibiting the PI3K/AKT signaling pathway, and induces G0/G1 cell proliferation and migration. (Thioridazine hydrochloride) inhibits the proliferation and migration of triple-negative breast cancer cells and induces apoptosis in G0/G1 cells after cycle blockade by inhibiting the PI3K/AKT signaling pathway [134]. Activation of D2/HIF-α signaling in response to stress stimuli promotes EMT and accelerates breast cancer and melanoma progression, while the D2 receptor antagonist trifluoperazine (TFP) inhibits tumor invasion and migration [135].	EMT immunosuppression Proliferation Migration	Thioridazine hydrochloride [134] TFP [135]
Lung cancer	D1	H727 bronchial carcinoid cells, A549 lung adenocarcinoma cells, H1299 lung large cell carcinoma cells, H292 lung mucosal epidermoid carcinoma cells.	D1 receptors inhibit non-small cell lung cancer progression by suppressing the activation of EGFR and ERK1/2, and SKF-38393 (a D1 receptor agonist) significantly inhibits cancer cell proliferation [136].	Proliferation	SKF-38393 [136]
	D2	A459 and NCI-H23 Human Lung Cancer Cells, Human Lung Cancer Tissue Sample, 4–6 Weeks Nude Mouse.	DA inhibits VEGF-induced proliferation and migration of HUVEC through the D2 receptor. The D2 receptor agonists quinpirole and Dostinex significantly reduced tumor growth [137]. activation of the D2 receptor inhibits the ERK1/2 and AKT signaling pathways and reduces Oct-4 and MMP-9 levels thereby significantly reducing the proliferation, clone formation and invasive ability of non-small cell carcinoma stem cells [138].	Proliferation Migration Invasion	Dostinex [137] Quinpirole [138]
Liver cancer	D1	MHCC97-H, MHCC97-L, SK-HEP-1, PLC/PRF/5, Huh-7, Hep-3B and Hep-G2 human hepatocellular carcinoma cells, MIHA human normal liver cell lines, and 6-week nude mice were constructed as xenograft tumor models.	D1 receptors promote tumor progression by regulating the cAMP/PI3KAKT/CREB pathway. SCH23390 significantly inhibited cancer cell proliferation and migration [139].	Proliferation Migration	SCH23390 [139]
	D2	Hepa1-6, H22 murine-derived hepatocellular carcinoma cells, SMMC-7721, BEL-7402 human-derived hepatocellular carcinoma cells, L02 human normal hepatocytes, 6-week-old male C57BL/6 mice as well as male thymus-less BALB/c nude mice. Hepa1-6 murine-derived hepatocellular carcinoma cells, AML12 liver parenchymal cells, C3H/HeN mice, C57BL/6 mice.	Moderate swimming enhanced DA levels in a mouse model of hepatocellular carcinoma, and DA inhibited the EMT process triggered by TGF-β1/Smad3 via the D2 receptor. Bromocriptine, a D2 receptor agonist, significantly reduced tumor volume and inhibited lung metastasis [140]. Domperidone, a D2 receptor antagonist, increased prolactin (PRL) levels and thus inhibited hepatocellular carcinoma progression. PRL inhibits TRAF-dependent innate immune signaling and c-Myc activity, slowing down hepatocellular carcinoma progression [141].	Proliferation Metastasis EMT	Bromocriptine [140] Domperidone [141]
Gastric cancer	D2	MKN28, SGC-7901, BGC-823 and MGC-803 Human Gastric Cancer Cells.	D2 receptor activation inhibits gastric cancer cell invasion and migration via the EGFR/AKT/MMP-13 pathway [142].	Invasion Migration	
Colorectal cancer	D2	HCT116 human colon cancer cells, BALB/c nude mice around 5 weeks old. HCT116 and SW480 human colon cancer cells, 5-6 months male BALB/c nude mice.	The D2 receptor antagonist Domperidone induces apoptosis by inhibiting the ERK/STAT3 pathway in human colon cancer HCT116 cells [143]. Pimozide (D2 receptor antagonist) is able to inhibit colorectal cancer tumor growth by suppressing the Wnt/β-catenin signaling pathway [144].	Proliferation Migration Apoptosis	Domperidone [143] Pimozide [144]
Cholangiocarcinoma	D1	NOZ, KKU213, TKF1 cholangiocarcinoma cells; human cholangiocarcinoma-derived organoids; 8-12 weeks NOD/SCID mice.	D1 receptor activation inhibits cholangiocarcinoma progression through the WNT signaling pathway [145].	Proliferation	
Melanoma	D2	B16 melanoma cells; 4–6-week maleC57BL/6 mice.	Endogenous dopamine inhibits tumor growth by inhibiting VPF/VEGF-mediated angiogenesis via D2 receptors on tumor endothelial cells [146].	Angiogenesis	
Glioblastoma	D2	Glioblastoma patient specimen; primary GBM cells and human normal astrocytes; 6-week male BALB/c nude mice. Glioblastoma patient tissue specimens; 5-week male BALB/c nude mice and C57BL/6J mice. U251 human glioma cell line; thymus-free nude mice, xenograft tumor model (GBM43, 12, 6, 5, and 39). U251, T98G, A172, and U87MG glioblastoma cells, HL60, HEL, K562 leukemia cells. Glioma patient tissue samples; thymus-free BALB/c and CD-1 nude mice.	D2 receptor antagonists induce glioblastoma death via death receptors 4/5 and by inhibiting MET activation [147]. Under chronic stress, DA and D2 receptors work together to promote glioblastoma progression through a positive feedback loop formed by the ERK/GSK3β/β-catenin pathway and ERK/TH, and the use of Pimozide, a D2 receptor antagonist, significantly inhibited tumor cell proliferation in vitro and growth in vivo [148]. The ability of the D2 receptor to induce HIF1α expression, increase the glucose uptake and glycolysis rate in GBM 39 cells [149]. Paired homology frame transcription factor (PRRX1) enhances self-renewal and differentiation of glioma stem cells through the D2 receptor-mediated ERK and AKT pathways, which in turn promotes tumor infiltration and metastasis [150]. The repressor element 1 silencing transcription factor (REST) regulates glioblastoma cell apoptosis through inhibition of the D2 receptor [151].	Proliferation Metabolism Metastasis Stemness Apoptosis	PPZ, ONC201 and ONC206 [147] Pimozide [148]

**Table 6:** Regulatory mechanisms of 5-HT receptors in cancer progression.

Cancer	Receptor	Model Materials	Mechanism	Tumor Impact	Targeted Therapy
Colorectal cancer	5-HT1	Tissue samples from human colon cancer patients; knockout mice and NSG mice on a C57BL/6 background.	Binding of 5-HT to the receptors 5-HT1B/1D/1F activates Wnt/β-catenin signaling and promotes tumorigenesis and metastasis induced by colorectal cancer stem cells CSC [175]. The 5-HT1A inhibitor Fluoxetine inhibits colon cancer progression by modulating NF-κB activation [176].	Proliferation Metastasis Stemness	GR127935 [177] Fluoxetine [176]
	5-HT2	CT26 mouse colon cancer cells; Cre mice, xenograft tumor models; human colon cancer tissue samples. HCT116, HT29 colon cancer cells; human colon cancer tissue samples. COLO-205 human colon cancer cells, CT25 mouse colon cancer cells; SNU-1235-co colon cancer organoids; 8-week male balb/c nude mice.	The 5-HT/5-HT2B/TGF- β signaling pathway plays a dual role in colitis related cancers: inhibiting tumorigenesis in the early stage and promoting tumor progression in the late stage [178]. 5-HT2B promotes colorectal cancer metastasis through EMT mediated by the creb1-zeb1 axis [179]. 5-HT2B inhibited colon cancer growth through ERK signaling. The 5-HT2B inhibitors GM-60186 and SB204741 significantly inhibited the proliferation and migration of cancer cells [180].	Proliferation Invasion Migration EMT	GM-60186 SB204741 [180]
Pancreatic cancer	5-HT1	PANC-1 and MIAPaCa-2 human pancreatic cancer cells, HPDE human normal pancreatic ductal epithelial cells. PANC-1, CFPAC-1 and other pancreatic cancer cells, HPDE6-C7 normal pancreatic ductal epithelial cells; xenograft tumor model.	5-HT1B/1D can promote the proliferation and migration of tumor cells by activating β 1-integrin-Src-FAK complex and TG2/NF-κB/EMT pathway [181]. 5-HT1D, as a key molecule of HOXA10-AS/miR-340-3p axis, reduces cancer cell apoptosis and promotes cancer cell proliferation and migration by regulating PI3K/AKT signaling pathway [182].	proliferation Invasion migration Apoptosis	
	5-HT2	PDAC cells, PDX xenograft models; human pancreatic cancer tissue samples; Pdx1-Cre, LSL-KrasG^12D/+^ and LSL-Trp53^R172H/+^ mice.	The 5-HT2B-LYN-p85 complex exacerbated the growth and metabolism of pancreatic tumors by activating PI3K/AkT/mTOR signaling and promoting Warburg effect, and SB204741 significantly slowed tumor growth [183].	Proliferation Metabolism	SB204741 [183]
Liver cancer	5-HT1	HCCLM3, Huh7 and other liver cancer cells, hepatocellular carcinoma tissue samples, xenograft tumor models. HepG2, SMMC-7721 hepatoma cells.	5-HT1D promotes liver cancer progression through the Wnt/β-catenin pathway. 5-HT1D interacts with PIK3R1 to activate the PI3K/AKT/FoxO6 pathway to promote liver cancer progression [184].	Proliferation Invasion Migration	
Gastric cancer	5-HT2	GES human normal gastric mucosal epithelial cells, AGS, HGC27, MGC-823, MKN-45, NCI-N87 gastric cancer cells; gastric cancer tissue samples; xenograft tumor model.	5-HT2B combined with Fyn regulates p85 activity, activates PI3K/AKT/mTOR signaling, inhibits iron death progression, and jointly promotes gastric cancer cell survival [185].	Proliferation Ferroptosis	SB204741 [185]
Prostate cancer	5-HT1	PC-3, Du145 and LNCaP human prostate cancer cells.	5-HT induces ERK1/2 and AKT activation through the receptor 5-HT1A, promoting cancer cell proliferation and migration [173].	Proliferation Migration	NAN-190 [173]
Breast cancer	5-HT1	LM2, 4173, MCF10CA1a, MDA-MB-231 and HS578T breast cancer cells, breast cancer patient tissue samples, female NOD-scid and BALB/c nude mice.	5-HT1A inhibits TGF- β downstream signals by interacting with TRIM21 and PSMD7, which in turn inhibits triple negative breast cancer progression [186].	Proliferation	
	5-HT2	MCF-7, MDA-MB-231 breast cancer cells, MCF10A non neoplastic breast epithelial cells.	5-HT promotes ERK1/2 activation, AKT phosphorylation, and HIF-α expression through 5-HT2A/2C receptor phosphorylation of JAK1 and STAT3, which together lead to increased PKM2, promotes glucose uptake, enhances mitochondrial metabolic oxidation, promotes breast cancer cell proliferation, and inhibits cancer cell apoptosis [187].	Proliferation Apoptosis Metabolism	ketanserin [187]
	5-HT7	MCF10A breast normal epithelial cells, MDA-MB-231, BT-546 and MCF-7 breast cancer cells. HCC-1395, T47D, HS578T human breast cancer cells; 6-week BALB/c nude mice.	5-HT via receptor 5-HT7/ FoxM1 signaling promotes breast cancer cell proliferation [188]. Breast cancer cells autocrine 5-HT acts on receptor 5-HT7, and then promotes invasion through Gα/cAMP, and proliferation through Gβγ/PI3K/AKT [189].	Proliferation Migration	Metergoline [188] BJ-113, SB269970 [189]
Melanoma	5-HT2	B16F10 mouse-derived melanoma cells.	Neuronal substance P promoted apoptosis in melanoma cell B16F10 by inhibiting 5-HT2A receptors [190].	Proliferation Apoptosis	ketanserin [190]
	5-HT3	WM-266-4, B16F10 mouse-derived melanoma cells.	5-HT3 antagonists promote melanoma cell apoptosis by inducing sub-G1 phase DNA accumulation and caspase-3 activation. In addition, they enhance intracellular Ca^2+^ levels, activate ERK1/2 phosphorylation, and inhibit NF-κB signaling, which in turn suppresses tumor progression [191].	Proliferation Apoptosis	tropisetron ondansetron [191]

## Data Availability

Not applicable.
